# The Protective Role of Type I Interferons in the Gastrointestinal Tract

**DOI:** 10.3389/fimmu.2017.00410

**Published:** 2017-04-06

**Authors:** Kevin P. Kotredes, Brianna Thomas, Ana M. Gamero

**Affiliations:** ^1^Department of Medical Genetics and Molecular Biochemistry, Temple University School of Medicine, Philadelphia, PA, USA

**Keywords:** interferon, intestine, inflammation, microbiome, epithelium

## Abstract

The immune system of the gastrointestinal (GI) tract manages the significant task of recognizing and eliminating pathogens while maintaining tolerance of commensal bacteria. Dysregulation of this delicate balance can be detrimental, resulting in severe inflammation, intestinal injury, and cancer. Therefore, mechanisms to relay important signals regulating cell growth and immune reactivity must be in place to support GI homeostasis. Type I interferons (IFN-I) are a family of pleiotropic cytokines, which exert a wide range of biological effects including promotion of both pro- and anti-inflammatory activities. Using animal models of colitis, investigations into the regulation of intestinal epithelium inflammation highlight the role of IFN-I signaling during fine modulation of the immune system. The intestinal epithelium of the gut guides the immune system to differentiate between commensal and pathogenic microbiota, which relies on intimate links with the IFN-I signal-transduction pathway. The current paradigm depicts an IFN-I-induced antiproliferative state in the intestinal epithelium enabling cell differentiation, cell maturation, and proper intestinal barrier function, strongly supporting its role in maintaining baseline immune activity and clearance of damaged epithelia or pathogens. In this review, we will highlight the importance of IFN-I in intestinal homeostasis by discussing its function in inflammation, immunity, and cancer.

## Introduction

The gastrointestinal (GI) tract has the greatest mucosal surface area of any organ system shared with the environment, interacting with a wide array of microbes and chemical irritants. These interactions with colonizing bacteria, especially early in life, are fundamental in developing proper gut health (1). The intestines of newborns are initially sterile (2), but become colonized immediately after birth, upon exposure to their new environment. The establishment of healthy intestinal microbiota can be hindered due to lack of exposure to commensal bacteria or upon treatment with antibiotic medications (3). This appears to be very important as there is mounting evidence that resident microbiota play an important role in shaping the function of the GI tract. The initiation and progression of human inflammatory bowel diseases (IBDs) are reliant on the dysregulation of complex interactions among genetic, environmental, and immune factors, as well as physical barriers within the intestinal mucosa. The physical barrier between the external environment and internal tissue is the first line of defense against microbial pathogens, toxins, and other environmental factors (4). This protective barrier is provided by the inner lining of the intestine, a single-cell layer of intestinal epithelial cells (IECs), and their specialized subtypes (e.g., Paneth, goblet, or enteroendocrine cells) (5). IECs serve an essential role as regulators of mucosal immune responses (6) and as cohabitants within the intestinal environs, which can be colonized by commensal or pathogenic bacteria, fungi, and viruses (7). Paneth cells, in particular, play a vital role in gut homeostasis (8–10) at least *via* expression of IFN-I and interferon-stimulated genes (ISGs) (11, 12). Ingested antigens and constituents of commensal bacteria are constantly testing the immune system of the gut. Responses to antigens can be either positive or negative, inducing an antigen-specific state of immunity (13). Cytokines like IFN-I act as initial signaling mechanisms within this innate immune system determining the durability and specificity of the response. Together, a series of direct responses and feedback loops are in place for maintaining gut homeostasis—preventing tissue damage, hyperplasia, malignancy, and ultimately cancer.

## Type I Interferons (IFN-I)

The innate immune system is a remarkable network that has evolved to protect the host against disease. It has the ability to detect a wide range of microbial markers and, in response, rapidly activate a number of inflammatory and antimicrobial pathways. Part of this sophisticated system involves the family of IFN-I (IFN-α or IFN-β). These immunomodulatory cytokines are broadly expressed as α-helical cytokines transcribed from 13 homologous IFN-α genes (IFN-α1 and -α13 are the same) and a single IFN-β gene (14). They play a critical role as first line of defense by promoting and shaping antiviral and antibacterial immunity. Constitutive, baseline expression of IFN-I is very low in the intestines, typical of most tissues (12, 15–18). IFN induction is a rapid event that can be triggered in response to viral attack (*via* recognition of cytosolic viral double-stranded RNA, 5′triphosphate single-stranded RNA, or viral DNA) and bacterial infections (*via* recognition of lipopolysaccharide, lipoprotein, or flagellin, for example) (19). Each response is activated by specific pattern-recognition receptors (PRRs), like RIG-like helicases and toll-like receptors (TLRs), expressed by different cell types (20). Secreted IFN-I then activates autocrine and paracrine signaling cascades *via* the heterodimeric IFN-I receptor complex (14). IFN-I bind to and activate the cognate cell surface receptor consisting of the IFNAR1 and IFNAR2 chains, which induce downstream signaling *via* tyrosine phosphorylation of JAK kinases (JAK1 and TYK2). Activated JAKs then phosphorylate the transcription factors STAT1 and STAT2 in the cytoplasm that in association with IRF9 from the heterotrimeric complex ISGF3. ISGF3 translocates to the nucleus and binds to the promoters of IFN target genes and activates the transcription of many ISGs (21). These ISGs drive immunomodulatory antiviral (22), antiproliferative (23), antibacterial (24), and antitumor actions (15) throughout the body, including the GI tract (18).

## IFN-I as Anti-Inflammatory Immunomodulators

Type I interferons not only function as signaling molecules of innate immunity but also promote the activation of adaptive immunity. It is well-established that systemic IFN-I can influence CD4^+^ T cell differentiation and function *via* their effects on dendritic cells (DCs). IFN-I drive DC activation and maturation (25), MHC II expression, and production of IL-12 (26, 27), to augment T helper (Th)1 cell responses. In addition, IFN-I can act directly on T cells to inhibit their expansion from lymph nodes, thus promoting DC–T cell interactions (28). Several studies also show that IFN-I enhance natural killer (NK), B, and CD8^+^ T cell activity (29, 30). By contrast, other studies present a different side of IFN-I—as key factors in the attenuation of an active immune response. Primarily, IFN-I increase the susceptibility of lymphocytes and macrophages to apoptosis (24, 31–34). IFN-I also inhibit the expression of IL-8, a chemotactic cytokine responsible for recruiting neutrophils and leukocytes to areas of inflammation (35, 36), and of IL-17, *via* inhibition of Th17 differentiation (37, 38). IFN-I antagonize the effects of local IL-17 by downregulating the expression of IL-1β, IL-23, and osteopontin, and by inducing the production of the anti-inflammatory cytokine IL-27 in DCs (38, 39). Induction of IFN-I in macrophages by bacterial infection reduces IL-17A/F variant expression, followed by a decrease in IL-17A(+) γδ T cells, further highlighting the role of IFN-I on T cell populations during infection (40). Further, IFN-I can inhibit the secretion of IL-1β, both by inhibiting production of pro-IL-1β and blocking pro-IL-1β cleavage to mature IL-1β *via* impeding inflammasome activation (41). To suppress inflammation, IFN-I also induce the secretion of anti-inflammatory cytokines (e.g., IL-10, IL-27, and IL-1RA) from phagocytes *via* expression of inhibitory feedback SOCS and PIAS proteins in T cells and phagocytes (42–44). Additionally, IFN-I suppress IFN-γ-induced MHC II expression by downregulating IFNGR1 levels as a negative feedback mechanism (45, 46), and high levels of IFN-I can inhibit IL-12 production during certain viral infections (47). IFN-I also inhibit inflammatory responses by inducing tristetraprolin, a strong suppressor of TNF-α and IFN-γ (48, 49).

Alterations to the IFNAR1 gene have been linked to susceptibility for IBD and changes to microbiome populations (50, 51), thus providing supporting evidence that IFN-I contribute to immune defenses against conditionally pathogenic microbiota and intestinal inflammation (52). In a T cell adoptive transfer model of colitis, signaling through host hematopoietic cell Ifnar1 was necessary to deter development of colitis. *Ifnar1^−/−^*-recipient mice developed severe colitis, compared with *Ifnar1^+/+^* mice, when inoculated with CD4^+^ T cells from a WT mouse (18). Phagocytes collected from the colonic lamina propria (LP) of *Ifnar1^−/−^* mice produced less IL-10, IL-1RA, and IL-27 than did cells from WT mice (18) demonstrating an important role for IFN-I signaling driving the expression of anti-inflammatory cytokines by gut phagocytes and maintenance of intestinal T cell homeostasis. Oral administration of the colonic irritant, dextran sulfate sodium (DSS) is another well-established model of acute colitis as it produces submucosal inflammation and ulceration in the gut thereby providing a “leaky” epithelial cell-lining ideal for translocation of luminal microbiota into the LP (53). *Ifnar1^−/−^* mice are found more susceptible to DSS-induced colitis pointing to conventional DCs as critical players in attenuating inflammation (16, 18, 54, 55). However, a later study found that deletion of *Ifnar1* in LysM^+^ myeloid cells, but not in conventional DCs exacerbated DSS-induced colitis (56). These differing results could be attributed to the mouse model employed. In the first study, Abe et al. used transgenic DTR mice with intact Ifnar1 to deplete CD11c^+^ DCs *via* administration of diphtheria toxin. By contrast, Rauch et al. used mice with conditional deletion of Ifnar1 in DCs or in myeloid cells. Nevertheless, both studies agree on the protective effect of IFN-I by suppressing IL-1 production during inflammation of the gut. Altogether, IFN-I activate and orchestrate different programs to keep inflammation under control.

## IFN-I are Instrumental in Maintaining Homeostasis in the Gut

Balance of the microbiome within the small and large intestine is important for not only maintenance of the intestinal epithelium, proper digestion, and nutrient uptake but is also strongly tied to immunity, inflammation, and cancer risk (57, 58). Both pro- and anti-inflammatory cytokines are chief among these immunomodulatory agents, including IFN-I, in regulating the growth and renewal of IECs (59–61). IFN-I are constitutively expressed in the intestines by LP CD11c^+^ DCs (16, 18, 62). The LP is the layer of connective tissue underneath the intestinal epithelium, enriched in immune cells. In the colon, CD11c^+^ DCs cells express mRNA for IFN-α5-, IFN-α9-, and IFN-I/ISGS3-induced genes thus indicative of active IFN-I production and signaling. Proper regulation of epithelial cell turnover in the intestinal lining is important for balance between replacement of damaged/sloughed cells and hyperplasia, which leads to pre-cancerous polyp formation (61, 63). Secretion of IFN-α has been shown as an important regulator of epithelial apoptosis. IFN-α administration prevented epithelial cell apoptosis in an *Escherichia coli*-induced mouse model of disease (64). Basolateral IFN-α also polarized monolayers of IECs, protected these cells against apoptosis, and promoted disruption of epithelial tight junctions (54). Moreover, IFN-α can induce the expression of GBP-1 (64), shown to prevent apoptosis, and promote intestinal epithelial barrier integrity (65). Prevention of apoptosis by IFN-α-induced GBP-1 subsequently inhibited endothelial cell angiogenesis (66, 67). In a study conducted in mice with deleted *Ifnar1* in the intestines, loss of IFN-I signaling increased the number of Paneth cells and hyperproliferation of epithelial cells with no signs of spontaneous inflammation or enhanced susceptibility to DSS, when compared to littermate controls (50, 51). Most recently, Fuchs et al. reported that increased protein levels of IFNAR1 *in vivo* [*via* deletion in the intestine of casein kinase 1α (CK1α), which controls the ubiquitination and degradation of both β-catenin and the IFNAR1] led to an increased ISG transcriptional signature (52) highlighting baseline IFN-I signaling in the intestinal epithelium. Deletion of CK1α in the intestines of *Ifnar1^−/−^* mice resulted in decreased levels of p21, inhibited p53 activation, and unrestricted IEC proliferation resulting in loss of gut barrier function and prompt animal death. Hence, IFN-I enable enhanced maturation, differentiation, and establishment of the cohesive epithelial barrier in the gut highlighting the contribution of IFN-I signaling to the control of IEC proliferation and function. As such, IFN-I are vital in maintenance of the host-microbiota equilibrium and constraining IEC proliferation and viability.

The microbiome in the gut plays an important role in the pathogenesis of IBD. This is evidenced by a variety of animal models in which development of intestinal inflammation is completely abolished under germ-free conditions (68). In healthy individuals, the gut microenvironment exists in a continuous state of controlled inflammation, despite the presence of potent antigen-presenting cells, like DCs. DCs are important for controlling T cell-mediated antigen response (69) and are the major source of TLR-driven IFN-I production (70). Conventional DCs have been attributed with inhibition of DSS-induced colitis, in part, to IFN-I production (14, 55). IFN-I regulated colonic recruitment of neutrophils and monocytes, as well as activation of pro-inflammatory macrophages (55). Additionally, *Ifnar1* loss in myeloid cells promoted colitis *via* increased IL-1 production (56), a pro-inflammatory cytokine produced by activated macrophages (71). Interestingly, in celiac disease (an IBD driven by strong T cell activation toward gluten), the role of IFN-I appears reversed. In humans, mucosal DC populations are increased in celiac disease patients (72). Activated mature DCs from these patients maintained higher IFN-α transcripts, as well as for IL-18 and IL-23, two cytokines responsible for Th1 polarization and subsequent IFN-γ production. Furthermore, IFN-α blockade inhibited IFN-γ transcripts in *ex vivo*-organ culture of celiac biopsy specimens challenged with gluten (72). Yet in mouse models of colitis pretreated with synthetic bacterial DNA, increased anti-inflammatory IL-10 and decreased IFN-γ production were reported (73). Along these same lines, a human ulcerative colitis (UC) study showed a correlation between IFN-I response and Th17 differentiation and suppression of IL-17 production (74). Th17 cells are central effectors that produce pro-inflammatory cytokines, particularly IL-17 in the gut (75, 76). IL-17 then induces the secretion of chemokines and antimicrobial peptides to create a mucosal barrier to eliminate pathogens; however, excessive IL-17 production exacerbates inflammation thereby promoting pathogen colonization (77).

T regulatory (Treg) cells play a central role in suppressing the development of intestinal inflammation and IBD (78–80). Tregs maintain intestinal homeostasis under conditions of continuous challenge with inflammatory microbes. Induction of Treg populations by recombinant bacterial DNA analogs was TGF-β- and IFN-I-dependent in a mouse model of IBD (81). Maintenance of the Treg population in the gut is mediated by IFN-I signaling driving the expression of Foxp3 in colonic Tregs (82). Continuous Foxp3 expression is necessary for the development and regulatory function of Tregs (83, 84). IFN-I limit inflammation by eliciting production of the regulatory cytokine IL-10 or by enhancing the activity of Treg cells (79, 85). Additionally, apoptotic resident intestinal DCs help regulate the populations of Tregs in the intestine *via* production of IFN-β (86). In IBD patients, Th1 and Th17 constitute a major driving force in the disease process in the inflamed mucosa characterized by high surface expression of activated CD69 (87, 88). Expression of CD69 is strongly induced by IFN-I (28). Several studies in mice indicate a role of CD69 in the regulation of arthritis (89), asthma (90), myocarditis (91), pathogen clearance (92), and tumor immunity (93). Commensal bacteria in the intestinal tract are shown to induce CD69 expression in CD4^+^ T cells. Secretion of the regulatory cytokine TGF-β1 by CD4^+^ T cells decreased, whereas production of the pro-inflammatory cytokines (IFN-γ, TNF-α, and IL-21) increased, upon deletion of CD69. CD69^−/−^ cells showed impaired IFN-β1 induction by TLR3 ligand polyI:C. CD4^+^ T cells lacking CD69 expression were hindered in their ability to mature into Tregs (Foxp3^+^) leading to accelerated colitis (94).

## IFN-I Confer Protection Against Colitis

Toll-like receptors play an important role in innate immunity by recognizing structurally conserved bacterial and viral components. TLRs are important transmembrane-signaling PRRs involved in inducing inflammation and are pivotal in the establishment of adaptive immunity. In addition to innate immune cells such as macrophages and DCs, IECs express a spectrum of TLRs (95). TLR signaling can induce strong production of several inflammatory cytokines, including IFN-I (96). TLR2 and TLR4 recognize bacterial cell wall components at the cell surface, while TLR3, TLR7, and TLR9 recognize bacterial or viral nucleic acids in endosomes after phagocytosis of bacteria or viruses (97). Activation of DCs *via* TLRs contributes to both rapid anti-pathogen responses and maintenance of homeostatic protective immunity (98). This is partly mediated by the direct production of cytokines necessary for the development of downstream humoral and cell-mediated immunity. Imiquimod, a TLR7 agonist, has been shown to ameliorate DSS-induced acute colitis by inducing the expression of IFN-I in the colonic mucosa (99). When administered as a preventive measure, ligands for TLR9 (CpG) or TLR3 (polyI:C) also induced IFN-I and lessened disease severity of DSS-induced colitis (54, 100). Administration of neutralizing antibodies against IFN-I also impeded these downstream anti-inflammatory effects *via* TLR9, thus highlighting the importance of IFN-I signaling in maintaining intestinal homeostasis and providing avenues for future therapeutics (54, 101). The activation of TLR9 by CpG dinucleotides initiates a cascade of innate and adaptive immune responses, at least partially mediated by secretion of IFN-I and IFN-γ, that results in cell-mediated Th1 and humoral immune reactions (102). The TLR9 signaling pathway can induce the production of inflammatory cytokines through nuclear factor κB and interferon regulatory factor (IRF)-5, and IFN-I through IRF7 (96). In other studies, comparison of transcriptome profiles from gnotobiotic mice, which lack commensal bacteria that constitute the microbiome, to three bacterial colonization models—specific pathogen-free mice, ex-germ-free mice with bacterial reconstitution at the time of delivery, and ex-germ-free mice with bacterial reconstitution at 5 weeks of age—showed that TLR-driven expression of *Irf3*, a crucial rate-limiting transcription factor in the induction of IFN-I, was essential for normal development of the host immune system (103). Commensal bacteria triggered the production of IFN-β *via* recognition of dsRNA by TLR3, which in turn protected mice from experimental colitis (104).

Inflammatory bowel disease is a group of intestinal chronic inflammatory conditions mainly UC and Crohn’s disease (CD) that affects part or the entire GI tract. The precise cause is unknown, but evidence overwhelmingly suggests symptoms arise from either pathogenic or commensal intestinal bacteria triggering an abnormal immune response. IFN-α-secreting DCs in gut-associated lymphoid tissues (GALTs) regulate differentiation of Tregs (105). GALTs are primary locations of host encounter with exogenous antigens and pathogens. Interaction of GALT with microbiota regulates both the size and duration of systemic immune responses (106, 107). The commensal microflora constituting the microbiome of the intestinal tract is strictly entwined in the well-being of the host. In particular, the balance of bacterial populations is directly related to IBD, though additional host-driven genetic predispositions are also suspected. Genome-wide association studies have implicated the locus containing IFNAR1 as a genetic risk factor for developing human IBD (50). In patients with IBD, chronic inflammation is a major risk factor for the development of GI malignancies (108). Patients suffering from IBD typically use non-specific medications to manage the symptoms and include steroids, 5-aminosalacylic acid derivative, immunosuppressants, or antibodies against TNF-α (109). Systemic administration of IFN-I to treat IBD patients has been evaluated and the results vary in suppressing disease burden (110–113). UC is associated with increased expression of IL-13 in NK T cells from the mucosa of the GI tract (114–116). IFN-I have been shown to deter IL-4/IL-13 transcription and secretion (117) by, as well as blocking of signaling in, human CD4^+^ T cells (118). In one small study, the majority of UC patients treated systemically with interferon-β-a1 showed reduced disease burden using rectal bleeding as a clinical measure. In the responder group, the clinical effect of IFN-I therapy correlated with decreased IL-13 production by LP mononuclear cells. By contrast, the non-responders had significantly higher production of IL-17 and IL-6 compared to responders (119). In cases where IFN-I therapy exacerbated the disease, parallel diseases in the patient may have complicated the correlated observations (111). Initial studies in an experimental model of colitis depicted the benefits of IFN-I in regulating intestinal growth, *via* apoptotic turnover of old cells or constitution of the hematopoietic cell population in the gut (54), but subsequent studies could not produce a therapeutic effect from IFN-I in IBD patients (120, 121). In an animal study, the therapeutic potential of IFN-β-secreting *Lactobacillus* (La-IFN-β) by delivering IFN-β in the gut prior to the induction of colitis was evaluated (122). Unexpectedly, this preventive measure heightened sensitivity to DSS when compared to mice pretreated with control *Lactobacillus*. Colitic mice that received La-IFN-β had increased intestinal secretion of TNF-α, IFN-γ, IL-17A, and IL-13 and decreased Treg populations in their small intestine. Intestinal DCs from La-IFN-β-treated mice and bone marrow-derived DCs exposed La-IFN-β showed decreased IFNAR1 expression. The underlying causes for the differing results of these various studies have yet to be identified. Further muddying the waters, conventional DCs can either enhance or inhibit DSS-induced colitis, independently of T cells, contingent on their manner of activation (55), emphasizing again the importance of IFN-I-driven immunoregulation in the gut.

## IFN-I in Colorectal Cancer

Like many other cancer types, colorectal cancer development has an inflammatory component. In fact, the risk of patients with IBD to develop CRC is strongly linked to the duration of the disease, anatomical extent, and severity of colonic inflammation (123). It is estimated that as much as 15% of all IBD patients will die of colitis-associated cancer (CAC), although early diagnosis and proper treatment of IBD symptoms can reduce the risk of CAC (124). IFN-I promote the recruitment and activation of tumor-parallel immune cells, the presence of which is believed to improve the prognostic pathological assessments of CRC (125). Aside from the immune-compartment-driven inflammation referenced earlier, genetic alterations within IFN-I signaling cascades have been implicated in CRC. To model CAC in rodents, the axozymethane (AOM)/DSS protocol was developed and is widely used to study colorectal cancer (126). Mice are given a single intraperitoneal injection of the carcinogen AOM, which is known to cause activating mutations in β-catenin, Kras and upregulation of Cox2, and iNOS (127). Addition of DSS given in multiple cycles generates a chronic inflammatory environment that reliably accelerates the carcinogenic effect of a single dose of AOM by dramatically shortening the duration of time for tumors to arise. Using this model, loss of Ifnar1 in IECs was reported to increase inflammation and severity of colitis. This poses cancer risk as evidenced by *Ifnar1*-expressing mice displaying decreased GI tumor burden corresponding with decreased mucosal inflammation (51). However, findings from our lab employing the same CAC model revealed a distinct and unexpected phenotype, in that loss of *Stat2* (an essential component in IFN-I signaling) reduced tumor burden and inflammation in the colon (128). To further establish the role of IFN-I in CRC, additional animal studies are warranted using the sporadic model of CRC, which also has a strong link to inflammation (129).

Another link to consider in CRC is the study of single-nucleotide polymorphisms (SNPs) in IFN-I-related genes that include STAT1, JAKs, IRFs, IFN-γ, and IFN-γR, which have been associated with increased CRC risk and disease progression (130, 131). In stark contrast, SNPs in IFNA7 and IFNA14 genes have been found associated with overall survival, more specifically in CRC patients without distant metastasis at time of diagnosis (132). These genes are located nearby several transcription factor-binding sites, but remains unknown how IFNA7 and IFNA14 directly influence overall survival, though they may still be regarded as potential CRC patient biomarkers. SNPs in IFNAR1 were also found associated with CRC risk (132), but how they affect IFN-I signaling and inflammation as a whole in the gut remains to be evaluated.

In humans, CRC tumor specimens show elevated mRNA expression of TLR9, IFNAR1, and IL-6, indicating that IFN-I-signaling components and effectors may be good predictors for overall survival (133). Other contrasting studies, however, find that TLR9 expression is decreased in hyperplastic and villous polyps from patients who develop CRC, further supporting a possible protective role for TLR9 expression against malignant transformation in colorectal mucosa (134). To add to the complexity of the role of signaling components of IFN-I and gene products of IFN-I, a recent study reported that *in vitro* formation of colorectal tumor spheroids, in the absence of IFN-I treatment, induced transcription of ISGs *via* IRF9/STAT2 (135). *In vitro*-tumor spheroids are characterized by non-proliferating, metabolically stressed cells in the hypoxic inner core, surrounded by actively proliferating cells in the outer layers. Knockdown of STAT2, but especially IRF9 inhibited accumulation of three ISGs: IFI27, IFITM1, and OAS1, whereas STAT1 knockdown had no effect. In addition, expression of IRF9 in this 3D model resulted in a significant decrease in the sensitivity of CRC cells to multiple chemotherapeutic drugs (135). Another ISG, ISG15, functions as a ubiquitin-like modifier, able to form covalent conjugates called “protein ISGylation” on many cellular proteins leading to a cellular stress response and increased inflammation. Elevated ISGylation has recently been proposed to promote intestinal inflammation and CAC in mice (136). In other studies, ATG16LI, which is not an ISG, has been found to regulate autophagy as well as innate immunity. A non-synonymous ATG16LI polymorphism carrying a T300A amino acid substitution is implicated in CD. Paradoxically, this SNP in CRC patients was found associated with increased overall survival and reduced metastasis. Data show an elevated IFN-I transcriptional signature and mitochondrial antiviral signaling suggesting that ATG16L1 T300A could be regulating IFN-I production (137). Further investigation is warranted to fully understand the contributions of IFN-I-related signaling pathways in CRC.

## Concluding Remarks

**Figure 1:**
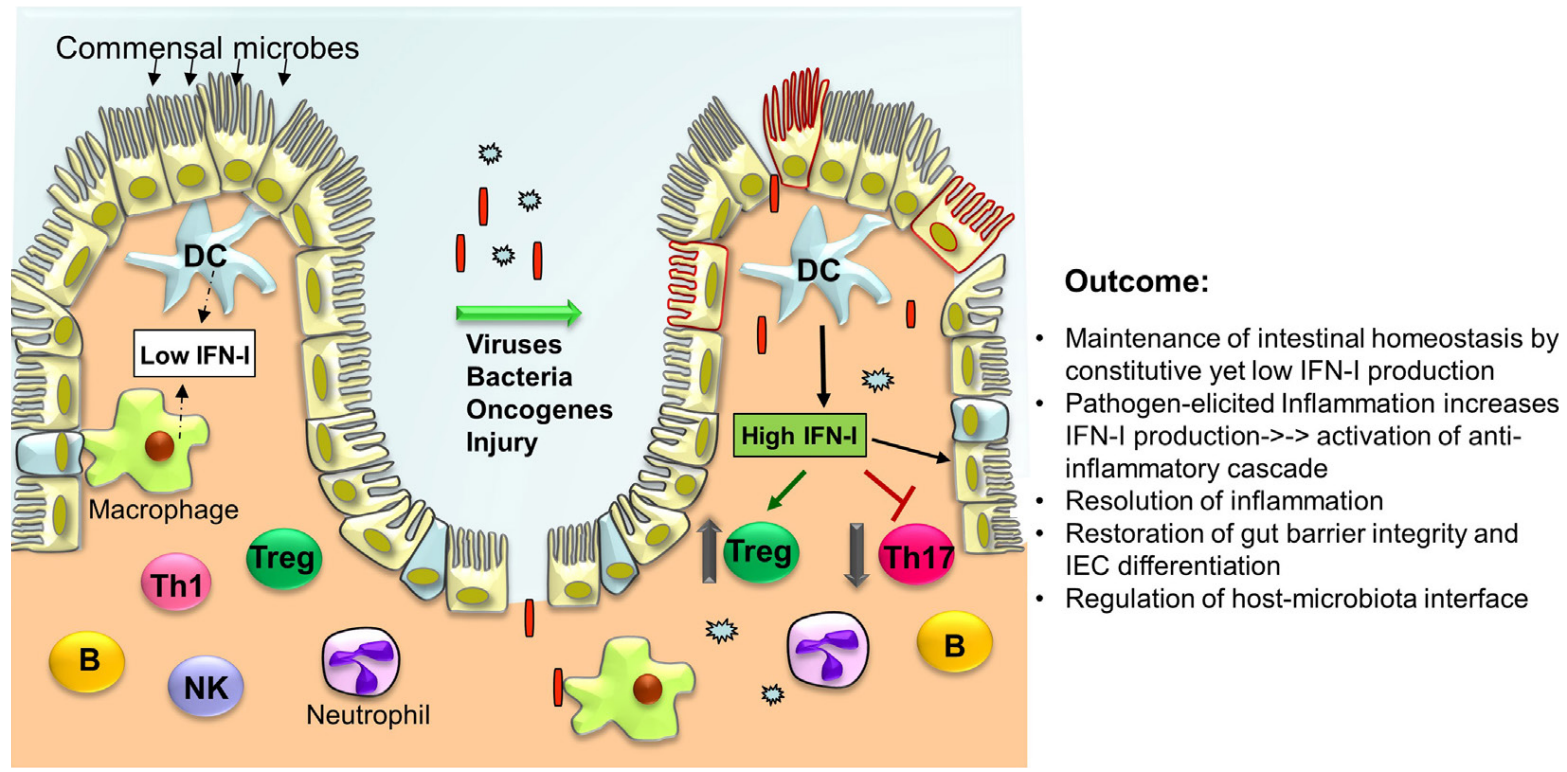
**Type I interferons (IFN-I) orchestrate a series of intracellular events in immune cells and intestinal epithelial cells (IECs) to cease inflammation resulting in the regeneration of the intestinal epithelium and restoration of the gut barrier**. Under normal conditions, low levels of IFN-I are secreted by lamina propria dendritic cells (DCs) and other phagocytes. In response to microbial attack and/or tissue injury, production of IFN-I by these cells is increased that in turn act on T cells to suppress Th17 cell differentiation while promoting Treg expansion thereby limiting inflammation. IFN-I also inhibit the production of pro-inflammatory cytokines (i.e., IL-1β, IL-8, IL-23) and induce the production of anti-inflammatory mediators (IL-1RA, IL-10, IL-27). Furthermore, IFN-I act on IEC and Paneth cells to restrict proliferation and favor their differentiation to establish gut barrier integrity.

Type I interferons are broadly expressed cytokines that drive innate immunity, responding to pathogenic attack or injury with both pro- and anti-inflammatory responses (summarized in Figure 1). This remarkable and well-orchestrated task is facilitated by the production of other cytokines and chemokines to eradicate the invading microorganism and begin the process of wound healing. IFN-I are the bridge between innate and adaptive immunity *via* promotion of DC maturation leading to disease-specific education and expansion of T cells. This process removes pathogenic microbes while promoting beneficial commensals. Similarly, IFN-I regulate epithelial cell apoptosis to promote intestinal barrier function. Deregulated immune responses to commensal bacteria that penetrate the intestinal epithelium barrier is believed to be the main cause of IBD, primarily UC and CD, which leave patients more vulnerable to CAC. Intensive research has been performed in experimental mouse models of colitis, however, to better understand the complex IFN-I-driven immunological effects, more studies are needed to better explain the diverse clinical results of IFN-I when evaluated in the setting of IBD. In fact, some patients appeared to respond better than others to IFN-I treatment, implying that additional factors must be identified to determine their regulatory role in IFN-I signaling. Most IECs appear capable of producing sufficient IFN-I, as well as other cytokines, and considering the important observations already made with *Ifnar1^−/−^* mice, the influences of STAT proteins and IFN-I-related proteins and parallel signaling pathways, will need to be taken into account and studied in more depth in future studies of intestinal immunity and homeostasis.

## Author Contributions

KK and AG wrote and edited the review. BT assisted with literature searches and organization of review.

## Conflict of Interest Statement

The authors declare that the research was conducted in the absence of any commercial or financial relationships that could be construed as a potential conflict of interest.
